# Basic, translational and clinical studies in reproductive medicine and clinical reproductive sciences

**DOI:** 10.1186/s12967-023-04108-4

**Published:** 2023-08-10

**Authors:** Fadi Choucair, Matteo Avella

**Affiliations:** 1 Reproductive Medicine Unit, Sidra Medicine, Doha, Qatar; 2 Maternal & Child Health Division, Sidra Medicine, Doha, Qatar

Infertility affects 10.7–15.5% of couples worldwide, and it is defined as the inability to conceive after 6–12 months of unprotected sexual intercourse [1]. Reproductive medicine is a fast-moving field whose ultimate goal is to provide people with better reproductive choices. Since the first human in vitro fertilization (IVF) assay [2, 3] and the first IVF pregnancies and children [4, 5], the demographic of individuals conceived through assisted reproductive technologies (ART) has been rapidly rising, getting close today to a 0.1% of the global human population [6]. ART includes medical procedures that involve the in vitro handling of gametes or preimplantation embryos to achieve pregnancy in patients seeking medical assistance.

Many research and clinical groups worldwide use various approaches to study, prevent, diagnose and treat reproductive disorders [7]. These tremendous research efforts have led to the development of several techniques regularly used today by fertility specialists. Although very diverse in nature, all these technologies share an essential commonality: their relevance to improving interventions and treatments in reproductive medicine. Since the seminal discovery of Intracytoplasmic Sperm Injection (ICSI) in 1992 [8], significant research efforts have focused on improving ART procedure outcomes. Examples include optimizing follicular stimulation protocols to obtain a sufficient number of quality ovulated metaphase II (MII) oocytes while preventing ovarian hyperstimulation syndrome [9]. Other examples are developing appropriate sperm selection strategies for patients requiring ICSI [10] and culture media and conditions to improve embryo development and maximize blastocyst formation [11] and embryo transfer for successful implantation [12]. In addition, for over two decades, preimplantation genetic testing (PGT) for aneuploidy (PGT-A) has been adopted to reduce the risk of miscarriage [13–15]. In addition, PGT for monogenic (PGT-M) or polygenic disorders (PGT-P) have been used to prevent the conception of embryos potentially affected by genetic diseases [16, 17].

Also, further advancements in computer and information sciences have been contributing to providing more targeted treatments in reproductive medicine [18], which include the selection of sperm [19] or blastocysts for embryo transfer [20]. At the same time, with the advent of Next Generation Sequencing tools and precise genome editing technologies, the genetics of infertility field has expanded significantly. Several studies have functionally characterized DNA variants affecting different aspects of human reproductive biology, which include sex determination, oogenesis and spermatogenesis, fertilization, and preimplantation embryo development [21–24]. The improved knowledge of aberrant mechanisms offers the promise of novel, effective and implementable medical interventions in the future [25, 26]. Moreover, although ART was initially designed to treat fertility disorders, as of today, these technologies are often used to provide patients with a diverse plethora of reproductive choices. For example, ART offers different fertility preservation options (e.g., gamete or embryo cryopreservation, ovarian tissue cryopreservation) in patients with the risk of gonadal failure due to chemotherapy or radiotherapy treatments [27].

We are delighted to launch a new section in the *Journal of Translational* Medicine named *“Reproductive Medicine & Clinical Reproductive Sciences*”. This section welcomes new research focusing on the basic mechanisms or the translational and clinical aspects of human reproductive biology. Also, we encourage the submission of preclinical and clinical studies whose results have the potential to prevent, diagnose, and treat reproductive issues, as well as studies that describe and characterize the genes and the mechanisms regulating human reproductive biology in cell lines or animal models. Reviews on existing and novel technologies, and commentaries on scientific breakthroughs in the field are also welcome. We look forward to receiving your contributions.
